# Copeptin Testing in Acute Myocardial Infarction: Ready for Routine Use?

**DOI:** 10.1155/2015/614145

**Published:** 2015-04-16

**Authors:** Sebastian Johannes Reinstadler, Gert Klug, Hans-Josef Feistritzer, Bernhard Metzler, Johannes Mair

**Affiliations:** Department of Internal Medicine III, Cardiology and Angiology, Medical University of Innsbruck, Anichstraße 35, 6020 Innsbruck, Austria

## Abstract

Suspected acute myocardial infarction is one of the leading causes of admission to emergency departments. In the last decade, biomarkers revolutionized the management of patients with suspected acute coronary syndromes. Besides their pivotal assistance in timely diagnosis, biomarkers provide additional information for risk stratification. Cardiac troponins I and T are the most sensitive and specific markers of acute myocardial injury. Nonetheless, in order to overcome the remaining limitations of these markers, novel candidate biomarkers sensitive to early stage of disease are being extensively investigated. Among them, copeptin, a stable peptide derived from the precursor of vasopressin, emerged as a promising biomarker for the evaluation of suspected acute myocardial infarction. In this review, we summarize the currently available evidence for the usefulness of copeptin in the diagnosis and risk stratification of patients with suspected acute myocardial infarction in comparison with routine biomarkers.

## 1. Introduction

The discovery of the biomarker cardiac troponin (cTn) as well as its introduction as a test into clinical routine has been one of the most important advances in the evaluation of patients with suspected acute myocardial infarction (AMI) over the last decades. Today, cTn plays a key role in the management of patients with acute coronary syndromes (ACS) [1]. A further clinically relevant increase in the sensitivity of cTn at an early diagnostic stage was achieved with the introduction of high-sensitivity (hs) cTn assays [2–4]. Despite these advances, there remains a troponin-blind period very early after symptom onset. Therefore, in patients with suspected AMI, a rule-out process with cTn measurement at presentation and 3 hours thereafter is still required when hs-cTn assays are used [5, 6]. Efforts to discover new biomarkers enabling a reliable earlier rule-out of AMI and thus a reduction of unnecessary hospital admissions are continuing. Besides its pivotal role as a diagnostic tool, cTn provides also information for risk assessment in the setting of ACS and many other cardiac and noncardiac diseases [5, 7–14]. Other promising biomarkers for risk stratification are natriuretic peptides (NPs) [7, 15, 16] and high-sensitivity C-reactive protein (hs-CRP) [17–19]. In the context of AMI, however, the incremental value of these biomarkers beyond conventional risk factors seems to be only moderate, and true large-scale comparative studies are still missing. Therefore, the role of novel biomarkers other than that of the routinely used cTn, NPs, and hs-CRP that might enable a better risk stratification of patients with chest pain is being increasingly investigated [20–25]. One of the most frequently proposed and extensively investigated biomarkers for facilitating the diagnosis of AMI is copeptin [6]. In addition, copeptin was also evaluated for risk stratification in this patient cohort. In this review, we will summarize the current clinical evidence for its routine use in patients with suspected AMI.

## 2. Pathophysiology of Copeptin

Located on chromosome 20, the gene named arginine vasopressin (AVP) encodes a 164-amino-acid peptide called pre-pro-AVP, which is produced by neurons of the hypothalamo-neurohypophysial system [26]. The mature pre-pro-AVP is the precursor molecule for AVP, which also includes a signal peptide, neurophysin II, and copeptin [27]. Copeptin (or C-terminal provasopressin) is a glycosylated 39-amino-acid peptide. As physiological function copeptin is believed to be involved in the proper folding of pre-pro-AVP [28, 29]. After transportation from the hypothalamus to the pituitary gland and cleavage of the pre-pro-AVP, copeptin is released into the circulation in stoichiometric amounts along with AVP. Both neuropeptides are primarily cosecreted in response to hemodynamic or osmotic alterations. The measurement of circulating AVP is challenging since AVP is an unstable molecule and because it is mainly bound to platelets [30–32]. Unlike AVP, copeptin is relatively stable in the circulation and methodologically easier to determine [31]. Therefore, copeptin is used as a surrogate marker for AVP release and an assay suitable for routine use has been developed [31].

**Figure 1:**
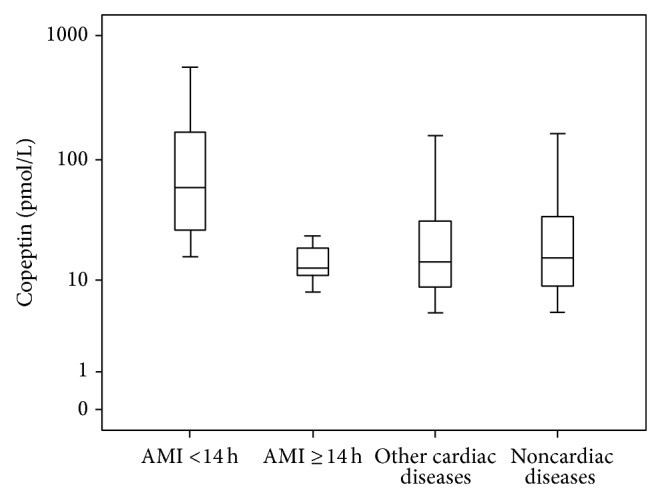
Distribution pattern of copeptin in patients with chest pain admitted to the emergency department (*n* = 171) according to discharge diagnosis. Our own unpublished data are shown as box plots. AMI patients were divided by delay from symptom onset. Copeptin concentrations in AMI patients presenting within 14 h from symptom onset were significantly (*P* = 0.013) higher than in the remaining patients, whereas AMI patients presenting thereafter did not differ significantly. Abbreviations—AMI: acute myocardial infarction.

In recent years, copeptin has been considered as a promising biomarker in numerous acute illnesses [33]. For instance, an association between elevated levels of copeptin and an unfavourable outcome were reported in patients with lower respiratory tract infections [34], sepsis [35], stroke [36], and acute pancreatitis [37]. These studies have consistently demonstrated a positive association between copeptin and disease severity. The role of copeptin has also gained particular attention in patients with AMI. Circulating copeptin levels are significantly higher during the acute phase in patients with AMI compared with healthy control subjects [38]. The copeptin elevation is again greater in patients with ST-segment elevation myocardial infarction (STEMI) than in patients with non-ST-segment elevation acute coronary syndrome (NSTE-ACS). The main trigger for copeptin release after AMI is thought to be acute endogenous stress [39, 40]. On the other hand, copeptin secretion is also associated with changes in fluid status [41]. Thus, it can be assumed that hemodynamic changes occurring in the acute phase during AMI might also trigger copeptin release [40]. In an animal study by Hupf et al., it was shown that vasopressin is also expressed in cardiac tissue [42]. Some authors speculate that myocardial necrosis could therefore directly lead to copeptin release from the heart [40, 43]. The release pattern of copeptin was recently described in detail [40, 43]. It is important to note that, in contrast to the MB isoenzyme of creatine kinase (CK-MB) and cTn, copeptin concentrations rise immediately after symptom onset and decrease rapidly thereafter (Figure 1). A direct association between the amount of released copeptin, on the one hand, and acute as well as chronic infarct size determined by cardiac magnetic resonance imaging, on the other, was demonstrated in STEMI patients [44, 45]. It is important to note, however, that, in contrast to cTn, increased copeptin levels are not specific for myocardial damage (see Table 1). For instance, Stallone et al. showed that increased levels of copeptin were measureable in about one-fifth of patients presenting to the emergency department with noncardiac chest pain [46].

## 3. Copeptin for the Diagnosis of Acute Myocardial Infarction

### 3.1. “Rule-In” of Acute Myocardial Infarction

As mentioned above, copeptin shows only low specificity for myocardial damage. Accordingly, the positive predictive value (PPV) for AMI of copeptin alone is thought to be unacceptably low. Indeed, the first studies investigating the diagnostic value of copeptin for AMI showed a very low PPV for AMI [47, 48]. For instance, Reichlin et al. calculated the PPV of copeptin for AMI diagnosis for different cut-off concentrations (9 pmol/L, 14 pmol/L, 20 pmol/L, and 24 pmol/L). In their study, the PPV of copeptin ranged between 34.9% and 57.9%. Two recently published meta-analyses confirmed that copeptin alone provides only insignificant diagnostic value in the setting of suspected AMI [49, 50]. Therefore, copeptin alone should not be considered as a single diagnostic marker in patients with suspected ACS.

### 3.2. “Rule-Out” of Acute Myocardial Infarction of Copeptin in Combination with Standard cTn and hs-cTn

**Figure 2:**
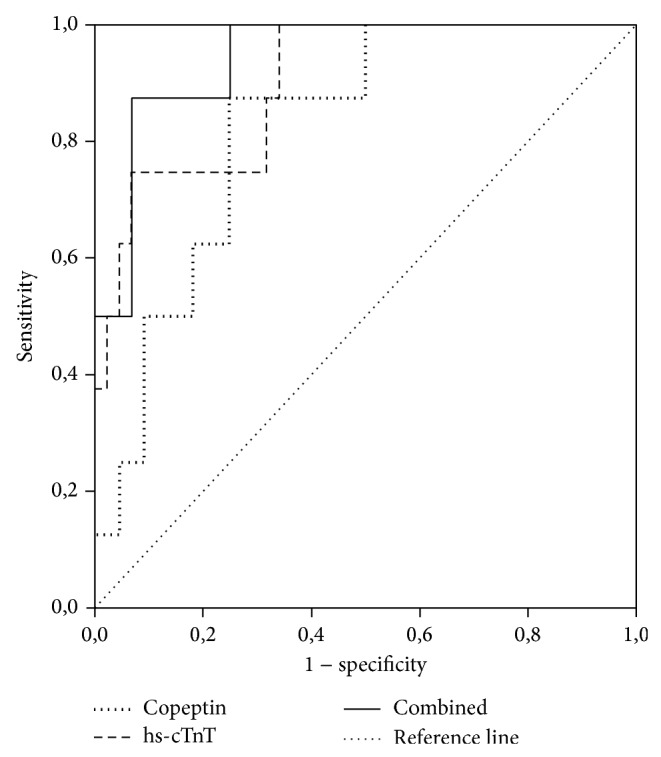
ROC analysis to compare the diagnostic power of copeptin, hs-cTnT, and the combination of both for the diagnosis of AMI in patients presenting with chest pain early after symptom onset (within 14 hours) on admission. Own unpublished data. The AUC of hs-cTnT (0.90, 95% confidence interval 0.79–0.97) did not differ significantly from the AUC of copeptin combined with hs-cTnT (0.94, 95% confidence interval 0.84–0.99; *P* > 0.05). Abbreviations—ROC: receiver operating characteristic; hs-cTnT: high-sensitivity cardiac troponin T; AMI: acute myocardial infarction; AUC: area under the curve.

On the basis of its unique release pattern, it was speculated that the combination of copeptin with cTn might facilitate the early “rule-out” of AMI. In fact, in the landmark trial published by Reichlin et al. in 2009, the authors concluded that the combination of copeptin and cTn enables a rapid and safe rule-out of AMI at presentation [47]. In their study, they investigated 487 unselected emergency department (ED) patients with symptoms suggestive of AMI. The combination of copeptin and cTn reached a sensitivity of 98.8% and a negative predictive value of 99.7% for ruling-out of AMI already at presentation. The combination performed significantly better compared to cTn alone. In addition, with the use of the dual marker strategy, the diagnostic accuracy was high for the diagnosis of AMI at presentation (area under the curve: 0.97). Nevertheless, some important limitations have to be mentioned. Although the cohort was comparable with other similar trials, it has all the limitations of a single-centre study. More importantly, a non-hs cTn assay was used, which was appropriate at that time but would not be today. Following this study, there have been a great number of reports confirming [48, 87–94] or rejecting [23, 95–99] this hypothesis. There are various reasons for these conflicting results. As mentioned before, one crucial point is the use of non-hs-cTn assay. hs-cTnT assays have been shown to provide a better early diagnostic sensitivity for AMI in the ED compared with the previous cTn assay generations [2, 100, 101]. Not surprisingly, almost all studies comparing copeptin with cTn assessed by a conventional assay display a substantial benefit of the dual marker approach. In contrast, when an hs-cTn is used, the benefit seems to be only moderate or absent [49] (Figure 2). The additive value seems to be especially low when the limit of detection (LoD) of hs-cTn is used as a decision limit for ruling out of AMI. This is of little surprise, since trials using LoD as “rule-out” criterion found a negative predictive value of up to 100% [102, 103]. On the other hand, a more sensitive assay for the determination of copeptin has been developed and is available for routine use as well [91]. This assay enables a more precise measurement of copeptin, which could also explain in part the differences in the “rule-out” studies. Moreover, initially performed studies used a copeptin cut-off value of 14 pmol/L, whereas recent data suggest that 10 pmol/L might be a more appropriate decision limit [49]. Another vital issue is the time point of copeptin sampling. As mentioned earlier, copeptin concentration increases to maximum immediately after symptom onset and decreases within hours thereafter. Hence, the proposed dual marker strategy is only reasonable in early presenters and when blood samples are drawn as early as possible. This fact might explain some negative studies when copeptin was measured with a notably delay after patient presentation [98].

### 3.3. “Rule-Out” of Acute Myocardial Infarction—Meta-Analysis and Interventional Trials

Meta-analyses performed so far have concluded that copeptin added to cTn results in significantly increased sensitivity for the diagnosis of AMI compared with cTn alone [49]. Of note, they also conclude that copeptin is most useful when combined with conventional cTn, and the added value remains uncertain when hs-cTn is used. The results of this meta-analysis, however, have to be interpreted with caution. Considerable heterogeneity among studies limits the interpretation of data. Furthermore, the literature search for this meta-anylisis was carried out in 2013. Important negative studies published in 2014 [95, 96] are therefore not included in analysis.

Probably the most important drawback of all above-mentioned studies is their observational design. In these studies, the measured copeptin value did not influence patient management. Management studies, however, showing that this novel approach is effective and safe are indispensable before copeptin can be recommended for routine use. Recently, the first and so far the only randomized, multicentre clinical process trial was published [104]. In this very important study by Möckel et al., 902 low-risk patients with suspected ACS were randomly assigned to either standard care (serial cTn testing as recommended) or an experimental arm. In the experimental arm, copeptin was measured in addition to cTn and if both biomarkers tested negative, the patient was eligible for discharge without the need for serial cTn testing. It needs to be mentioned that, in the latter case, the treating physician could still decide not to discharge the patient immediately. The primary safety study end-point was defined as combination of all-cause death, survived sudden cardiac death, AMI, rehospitalization for ACS, acute unplanned percutaneous coronary intervention, coronary artery bypass grafting, and documented life-threatening arrhythmia 30 days after enrollment. The authors found a similar event rate (standard arm: 5.17%, experimental arm: 5.19%) in both study arms, suggesting that the combined cTn/copeptin strategy is as safe as the standard procedure. The advantage of the new approach was also clearly demonstrated. In the experimental arm, hospital stay was significantly shorter compared with standard arm (4 versus 7 hours, resp., *P* < 0.001). The main conclusion was therefore that a dual marker strategy with copeptin and cTn could safely decrease length of hospital stay. Although the study was well designed, there are some relevant limitations that have to be considered. cTn was measured by different conventional (three sites) as well as high-sensitive assays (four sites) across the study sites. Furthermore, the thresholds for a “negative” cTn result were defined by the 99th percentile upper reference limit. By using the LoD as cut-off, the negative predictive value of cTn can be further increased as demonstrated by recent studies [103]. In the era of hs-cTn assays, a crucial study would be the direct of comparison of hs-cTn versus copeptin plus hs-cTn with the LoD as a rule-out cut-off for hs-cTn. Whether copeptin still provides an incremental value in this case remains to be investigated.

### 3.4. “Rule-Out” of Acute Myocardial Infarction in Point-of-Care Testing

In times of overcrowding of the emergency departments, point-of-care testing (POCT) becomes increasingly appealing. Recent evidence suggests that POCT might allow a fast and accurate diagnosis of AMI [105]. Nowadays, cTn is the most efficient diagnostic marker also in POCT [105]. Because of the lower sensitivity of POCT, cTn assays [106] compared to assays used in the central laboratory and the early period of “troponin blindness” novel biomarkers might improve the early diagnostic sensitivity in POCT. The use of the CK-MB and myoglobin was recently tested but failed to improve diagnostic performance [105]. The different release kinetics of copeptin compared with cardiac troponin after AMI makes copeptin a very promising candidate for POCT [40]. Furthermore, the encouraging results for the combination of copeptin with standard troponin assays used in central laboratory analysis suggest that a significant improvement might be possible also in POCT. Till now, prospective randomised trials are missing, but a dual marker POCT strategy including copeptin and cTn is worth being prospectively evaluated in future studies.

## 4. Copeptin for Risk Stratification in Acute Coronary Syndromes

In order to optimize timing and intensity of therapeutic interventions as part of patient management, adequate risk stratification at an early stage after ACS is necessary [5]. Early echocardiography data revealed that copeptin concentrations, assessed 3–5 days after AMI, are correlated with left ventricular dysfunction as well as remodelling 5 months after the event [107]. More recent cardiovascular magnetic resonance studies confirmed the association between copeptin, myocardial function, and adverse remodelling following STEMI treated with primary coronary intervention [44, 45]. Interestingly, the combination of day 2 copeptin and NT-proBNP levels could exclude the development of adverse remodelling over 4 months after AMI. One might therefore speculate that STEMI patients with increased copeptin concentrations after revascularization might benefit from more intense therapeutic regimens [108].

Whether copeptin is of prognostic value among patients with AMI was studied for the first time by Khan et al. in the Leicester Acute Myocardial Infarction Peptide (LAMP) study [38]. In this single-centre study of 980 consecutive patients with AMI, increased values of copeptin (odds ratio: 4.14, *P* < 0.0005) measured between days 3 and 5 after AMI were associated with the primary end-point of death or heart failure at 60 days in multivariate analysis. The area under the curve (AUC) for the prediction of the primary end-point for copeptin (0.75) was similar to that of NT-proBNP (0.76). Interestingly, the combination of both biomarkers led to a significant improvement of AUC (0.84), suggesting that a dualmarker strategy might be more useful for risk assessment in patients suffering an AMI. Because approximately 80% of the included patients in LAMP had a STEMI, a second study with only non-ST segment elevation-ACS (NSTE-ACS) patients was performed by the same study group [109]. The LAMP II study showed in 754 NSTE-ACS patients that copeptin (measured within 24 hours after admission) is an independent predictor of all-cause mortality at 6 months. In contrast to NT-proBNP, copeptin yielded a significant net reclassification improvement of 13% (*P* = 0.008) when added to the GRACE score.

The prognostic utility of copeptin levels on admission to hospital in patients with suspected AMI was recently documented in a prospective, single-centre study by Afzali et al. [110]. In this study of 230 patients, 107 had the final diagnosis of AMI (24 STEMI and 83 NSTE-ACS). The authors showed that levels of copeptin on admission significantly predict 180-day mortality. The AUC of copeptin (0.81) was higher compared with the AUC of cTnI (0.76) and the combination of both biomarkers (0.83) performed again better than either marker alone. Although copeptin measured at admission in 377 NSTE-ACS patients was related to death within one month after the index event, this association did not remain significant after adjusting for baseline characteristics or cTn levels in the COPED-PAO study [111].

To further elucidate the value of biomarkers in the post-AMI risk assessment, a recently published large study by O'Malley et al. compared the prognostic performance of multiple biomarkers sampled at enrollment among 4,432 prospectively recruited subjects with NSTE-ACS [20]. The authors conclude that, although cTn-I performed best among all investigated outcomes, copeptin seems a robust prognosticator for cardiovascular death and heart failure beyond established biomarkers. Therefore, copeptin appears promising for improving risk stratification in conjunction with other biomarkers. Of note, the authors could confirm previous data from the LAMP study indicating that copeptin is less suited to predict recurrent ischaemia. This might be explained by the fact that copeptin is primarily released in response to hemodynamic stimuli, but not by progression of atherosclerosis. A meta-analysis published in 2014 showed that the predictive value of copeptin and cTn for all-cause mortality is the same [49].

A relevant limitation of the above-mentioned studies is that none of these compared copeptin with hs-cTn. One study investigating the combination of these two biomarkers was recently published [112]. Patients with preexisting coronary artery disease and symptoms indicating AMI (*n* = 433) were analysed in a prospective multicentre fashion. Copeptin determined on admission provided prognostic information for the risk of death at 1 year after enrollment. More importantly, the combination of copeptin with hs-cTn yielded significantly enhanced prognostic accuracy. Further investigations are warranted to confirm these promising data.

A further important question is the time point of copeptin testing. Great between-study heterogeneity exists regarding the time point of copeptin sampling. Studies measuring copeptin at different time points during the (sub-) acute phase after ACS are lacking and therefore the optimal time point for assessing copeptin concentration remains unknown.

Another major drawback of the currently available evidence is that no study evaluated a copeptin (or multimarker) based therapeutic decision pathway. Prospective interventional trials are warranted to elucidate if measurement of copeptin provides additional information beyond established risk tools that impact treatment decisions which might improve patient outcome.

## 5. Summary

For the diagnostic evaluation of AMI, cTn remains the “gold standard” biomarker. There is enough evidence from observational studies indicating that a dual marker strategy combining measurments of copeptin and cTn levels using a conventional assay might facilitate the “rule-out” of AMI in early presenters. Also in POCT, such a dual strategy seems promising, but randomized clinical trials are lacking. However, when hs-cTn assays are used, the advantage of this approach seems insignificant. Data from a first randomized, controlled clinical process trial are promissing as they suggest that this new strategy allows early and safe discharge, but further prospective interventional trials are needed to confirm those results for the combination of copeptin with hs-cTn. Real-world data from large regrities are also necessary to accurately evaluate this strategy. Therefore, based on the currently available body of evidence, we do not believe that copeptin testing can yet be recommended for use in routine clinical practice if hs-cTn assay is used.

For prognostic evaluation, current data support the use of copeptin, integrated into a multimarker approach, to improve the classification of AMI patients into different risk groups early after the acute event. However, studies showing that a biomarker-guided strategy for risk stratification improves patient outcome are needed before testing for copeptin (and other biomarkers) can be recommended for implementation in clinical routine.

## Figures and Tables

**Table 1:** Overview of clinical conditions other than AMI associated with increased copeptin concentrations.

Condition	Potential implications of elevated copeptin concentrations	References
Stable coronary artery disease	Predictor for major adverse cardiovascular events	[51]
Heart failure	Associated with mortality risk, risk of hospitalization, and disease severity	[52–56]
Type 2 diabetes	Potential marker for peripheral arterial disease and diabetic chronic kidney disease. Potential marker for cardiovascular and all-cause mortality	[57–59]
Pneumonia	Marker for adverse outcome	[60, 61]
Acute exacerbation of chronic obstructive pulmonary disease	Potential prognostic marker for short-term and long-term outcome	[62]
Sepsis/shock	Promising independent prognostic markers for mortality	[33, 35, 63]
Survivors of cardiac arrest	Potentially useful for risk stratification at the time of hospital admission	[64]
Pulmonary arterial hypertension	Potentially useful in the prediction of poor outcome	[65]
Stroke/transient ischaemic attack	Risk stratification for patients with transient ischaemic attack and stroke	[66–68]
Traumatic brain injury	Probable marker of progressive haemorrhagic injury, acute traumatic coagulopathy, and mortality	[69–71]
Intracerebral haemorrhage	Useful to predict adverse clinical outcomes	[72, 73]
Carotid endarterectomy	Probable predictor of perioperative stroke	[74]
CABG surgery	Postoperative copeptin concentrations might predict delirium and cognitive dysfunction	[75]
Chronic kidney disease	Potential marker for the development/progression of atherosclerosis	[76]
Autosomal dominant polycystic kidney disease	Potential role in disease progression	[77, 78]
Carbon monoxide poisoning	Associated with intoxication severity and potentially useful to predict delayed neurological sequelae	[79]
Polycystic ovary syndrome	Relationship with cardiometabolic parameters (e.g., carotid intima media thickness)	[80]
Endometriosis	Direct association with disease severity	[81]
Preeclampsia	Associated with increased risk for preeclampsia already before clinical diagnosis	[82, 83]
Acute pancreatitis	Marker for disease severity and local complications	[37, 84]
Liver cirrhosis	Associated with the severity of disease and with the risk of death or liver transplantation	[85]
Sickle cell anaemia	Differentiation between mild or severe sickle cell anaemia	[86]

Aortocoronary bypass grafting (CABG).
